# Excellent Response to Plasma Exchange in Three Patients With Enterovirus-71 Neurological Disease

**DOI:** 10.3389/fneur.2019.00548

**Published:** 2019-05-24

**Authors:** Elba Pascual-Goñi, Maria Josa, Cristian Launes, Luis Querol, Marga del Cuerpo, M. Alba Bosch, Iolanda Jordan, Eulàlia Turón-Viñas

**Affiliations:** ^1^Department of Neurology, Hospital de la Santa Creu i Sant Pau, Barcelona, Spain; ^2^Department of Pediatrics, Hospital de la Santa Creu i Sant Pau, Barcelona, Spain; ^3^Pediatric Infectious Diseases Research Group, CIBER of Epidemiology and Public Health (CB15/00067 Group), Institut de Recerca Pediàtrica Sant Joan de Déu, Esplugues, Spain; ^4^Department of Microbiology, Hospital de la Santa Creu i Sant Pau, Barcelona, Spain; ^5^Banc de Sang i Teixits, Hospital de la Santa Creu i Sant Pau, Barcelona, Spain; ^6^Pediatric Intensive Care Unit, Hospital Sant Joan de Déu, Esplugues de Llobregat, Spain

**Keywords:** enterovirus, EV71, encephalomyelitis, brainstem encephalitis with cardiorespiratory failure, plasma exchange, immunotherapy

## Abstract

The clinical spectrum of Enterovirus-71-associated neurological disease includes acute flaccid paralysis, encephalomyelitis, or brainstem encephalitis with autonomic dysfunction. As no specific antiviral treatments are available, intravenous human immunoglobulin is used in early stages of the illness, decreasing serum proinflammatory cytokines, and improving clinical outcomes. Plasma exchange aims to eliminate pathogenic autoantibodies and proinflammatory cytokines, and is used in diverse immune-mediated neurologic conditions. However, its effect in Enterovirus-71 infections is unknown. We report three cases of severe Enterovirus-71 neurological disease treated with plasma exchange during an outbreak in Catalonia (Spain) in 2016. We observed a striking improvement in all three patients within 48 h of starting plasma exchange. Patients received four to six sessions every other day. Good outcomes were confirmed at the 1-year follow-up visit. Our observations suggest that plasma exchange is an effective complementary therapy for severe Enterovirus-71 neurological disease.

## Introduction

Enterovirus-71 (EV-71) belongs to the human enterovirus A species of the Picornaviridae family. It is an emergent pathogen responsible for a broad spectrum of infectious syndromes. EV-71 is a well-known cause of hand, foot and mouth disease (HFMD) in children, and is usually a self-limiting infection (1). Rarely, some patients develop severe neurological disease, defined as the presence of acute flaccid paralysis, encephalomyelitis, or brainstem encephalitis with autonomic dysfunction (2) that may be fatal in 7% of patients (3). Since EV-71-associated neurologic disease was first described in California in 1969 (4), several outbreaks have since been reported in the Asia-Pacific region and in Europe. An outbreak of brainstem encephalitis and encephalomyelitis occurred in Catalonia in 2016, and involved a new recombinant strain: EV-71 subgenogroup C1 (5, 6). This strain is highly neurotropic, and neurological involvement is often present.

No specific antiviral treatment has shown any benefit to date. Since EV-71 (7) has been associated with a cytokine-mediated inflammatory response, patients with severe neurologic involvement receive immunomodulatory treatments such as intravenous human immunoglobulin (IVIg). When administered in the early stages, IVIg may improve outcomes by decreasing plasmatic cytokines (2). Despite current treatments, however, neurological deficits remain in around 10% of patients (3). For this reason, additional therapeutic strategies are needed. In this report, we describe three cases of EV-71-associated severe neurological disease that showed a clear clinical improvement following treatment with plasma exchange (PEX). We postulate that PEX could be a complementary approach to eliminate the humoral factors and modulate the inflammation involved in the pathogenesis of the EV-71 neurological disease.

## Case reports

We report three patients (Table 1) who presented EV-71 severe neurological disease (two infants with brainstem encephalitis with cardiorespiratory failure and one adult with encephalomyelitis) during the outbreak in Catalonia (Spain) between April and June 2016 (5, 6). Clinical and MRI findings were compatible with encephalomyelitis. Enterovirus RNA was detected by polymerase chain reaction (PCR) in stool and throat specimens, and EV-71 was isolated in cell cultures. Diagnosis, disease severity stratification and treatment were conducted according to local (8) and WHO guidelines (2). Due to the disease severity and rapid neurological deterioration despite standard treatment, all three patients were treated with PEX. During each PEX session, 1 to 1.5 total plasma volume was removed and replaced with albumin. The procedure was performed every other day. Motor and cognitive outcomes were assessed and modified Rankin Scale score (mRS) was obtained at 3- and 12-month follow-up.

**Table 1:** Clinical and laboratory features of patients.

	**Patient 1**	**Patient 2**	**Patient 3**
Age	9 months	8 months	37 years
Sex	Male	Male	Male
Past medical history	None	None	Smoker
Symptoms/signs	Lethargy (GCS = 10), bulbar palsy, tetraparesis, and hypercarbic respiratory failure	Lethargy (GCS = 10), viral exantema, bulbar palsy, tetraparesis, and hemodynamic instability	Headache, aphasia, focal motor seizures, tetraparesis, and urinary retention
EV71 isolation	throat and rectal swabs	throat and rectal swabs	rectal swabs
CSF cell count	44 cells/mm^3^ (90% lymphocytes)	100 leucocytes/mm^3^ (95% lymphocytes)	22 cells/mm^3^ (93% neutrophils) Negative oligoclonal bands
MRI	T2- hyperintensities in dorsal brainstem and cervical spinal cord	T2- hyperintensities in dorsal brainstem and cervical spinal cord	Left frontal cortical thickening, leptomeningeal enhancement, cervical, and lumbosacral lesions with contrast enhancement
Diagnosis	Brainstem encephalitis with cardiorespiratory failure plus myelitis	Brainstem encephalitis with cardiorespiratory failure plus myelitis	Encephalomyelitis
Conventional treatments	IVIg, methylprednisonlone, milrinone	IVIg, methylprednisonlone, milrinone	No
Time from neurological disease onset to start PEX	48 h	7 days	72 h
Number of PEX sessions	6	4	5
Time from PEX to clinical improvement	12 h	48 h	24 h
Outcomes (1-yr follow-up)	Asymptomatic (mRS = 0)	Mild axial hypotonia (mRS = 1)	Asymptomatic (mRS = 0)
3-month Follow-up MRI	Mild T2-hyperintensity in dorsal brainstem, disappearance of the cervical lesion	Persistent lesions in dorsal brainstem and anterior cervical spinal cord	Brain MRI: Resolution of previous lesions

*CSF, cerebrospinal fluid; GCS, Glasgow Coma Scale; IVIg, intravenous human immunoglobulin; mRS, modified Rankin Scale score*.

## Patient 1

**Figure 1:**
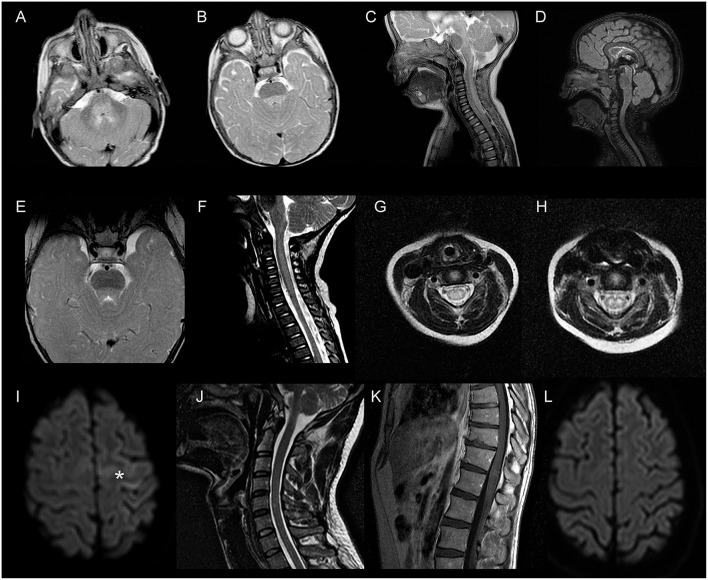
Brain and spinal cord MRI findings. Patient 1 – **(A–C)**: dorsal brainstem and cervical hyperintensities on T2-weighted images. **(D)**: sagital FLAIR image shows disappearance of previous spinal cord hyperintensity. Patient 2—**(E–G)**: dorsal brainstem and cervical diffuse and poorly defined T2-hyperintensities during the acute phase; **(H)**: hyperintense T2 signal of the anterior horn of the cervical spinal cord one week later. Patient 3—**(I–K)**: left frontal cortical hyperintensities (asterisk) in diffusion weighted images (DWI); cervical and lumbosacral hyperintensities on T2-weighted imaging with enhancement on post-gadolinium image. **(L)**: axial DWI at 3 months shows resolution of the previous alterations.

A 9-month-old infant presented with lethargy and respiratory failure. He had been diagnosed of herpangina 4 days before admission. Initial examination revealed bulbar palsy and flaccid tetraparesis. Brain and spinal cord MRI (Figures 1A–C) showed dorsal brainstem and cervical T2-hyperintensities. CSF revealed pleocytosis, and enterovirus PCR was negative. EV-71 was isolated in throat and rectal specimens. Although methylprednisolone (30 mg/Kg/day, for 3 days) and IVIg (1 g/Kg/day for 2 days) were started, a few hours later he developed high fever, hemodynamic instability, and respiratory failure requiring mechanical ventilation. Due to the dramatic evolution, PEX was started 48 h after onset. Twelve hours later, a marked clinical response was observed and he was extubated after a second PEX session. Six PEX sessions were performed every other day. He continued to improve and no significant complications were observed. Two weeks later, he presented intention tremor that had disappeared at 3- and 12-month follow-up (mRS = 0).

## Patient 2

An 8-month-old infant with a viral exanthema presented with lethargy and hemodynamic instability requiring mechanical ventilation. An echocardiography showed reduced ejection fraction (40%). Brain and a spinal cord MRI (Figures 1E–G) demonstrated dorsal brainstem and cervical diffuse and poorly defined T2-hyperintensities, compatible with encephalomyelitis. CSF showed pleocytosis and enterovirus PCR was negative. EV-71 was isolated in throat and rectal specimens. Methylprednisolone (30 mg/Kg/day, for 3 days), milrinone and IVIg (1 g/Kg/day for 2 days) were started at admission. Five days later he was extubated and examination revealed bulbar palsy and upper limb hyperreflexia. These symptoms improved over the following week. He then presented with myoclonus, decreased movements and lethargy, leading to reintubation. Methylprednisolone (30 mg/Kg/day, for 3 days) and IVIg (1 g/Kg/day for 2 days) were restarted and a new brain MRI showed persistent brainstem T2-hyperintensities in dorsal brainstem. On the other hand, hyperintense T2 signal of the anterior horn of the spinal cord was also observed (Figure 1). Given the worsening of symptoms, PEX therapy was started, and four sessions were performed every other day. The consciousness status, muscular tone and motor function improved notably from the second day. Two weeks later he had axial hypotonia with lack of head control and tetraparesis (4/5 on the Medical Research Council scale in all limbs) with hyperreflexia and mild hypertonia. During the follow-up a progressive improvement of the tetraparesis was observed. At 3-month follow-up, mild hypertonia of the right arm and lack of full head control persisted (mRS = 3). At 12 months, cognitive and motor functions were normal except for mild axial hypotonia (mRS = 1).

## Patient 3

A 37-year-old male, admitted to our hospital with severe headache, aphasia, focal motor seizures, and progressive tetraparesis. The previous week he had developed fever and oral ulcers. Two weeks earlier, his 3-year-old son had had a febrile exanthema and EV-71 had been isolated in the child's stool. Neurological examination of patient 3 revealed motor aphasia, right facial paralysis, flaccid tetraparesis and hyperreflexia. Brain MRI (Figure 1I) revealed a left frontal cortical thickening in fluid-attenuated inversion recovery (FLAIR) images, hyperintense in diffusion weighted images (DWI), with leptomeningeal enhancement. Spinal cord MRI (Figures 1J,K) revealed cervical and lumbosacral lesions. CSF showed pleocytosis and enterovirus PCR was negative. Enterovirus RNA was detected by PCR in stool specimens. Given the rapid deterioration and the signs of inflammation in the MRI, PEX therapy was initiated 72 h from the onset of the neurological symptoms. A striking clinical improvement was observed within 24 h after first session. Five sessions of PEX were performed every other day and no complications were observed. At 3- and 12-month follow-ups he was asymptomatic (mRS = 0).

## Discussion

We report three cases of severe neurological disease associated with EV-71 that responded to plasma exchange despite poor response to conventional treatment. A clinical response was observed within 24–48 h after the first PEX session. We did not detect any relevant side effects associated with this procedure. The positive response to PEX suggested a role of a deleterious inflammatory response in the pathophysiology of these syndromes.

All three patients presented serious EV-71 complications, with autonomic dysfunction in two cases. Autonomic dysfunction is of major concern in EV-71 neurological disease. In such cases, it has been hypothesized that cytokine release injures the vasomotor center of the brainstem, increasing pulmonary vessel permeability and leading to pulmonary edema (9). Interestingly, Shao et al. (10) showed that levels of proinflammatory cytokines and chemokines (except IL-8 and IL-4) in plasma were higher in patients with encephalitis plus cardiorespiratory failure than in those with encephalitis alone. Given the role of cytokines in EV-71 neurologic disease, several immunomodulatory treatments have been used, with varying results. Steroids, for example, have been used extensively in viral and inflammatory myelitis (11) and in EV-71 neurologic disease (3), but little is yet known about their benefits and mechanisms of action. Milrinone has been used in cases of autonomic dysfunction due to its anti-inflammatory effect and its well-known inotropic properties (12). And third, IVIg has been used in EV-71 neurologic disease according to WHO guidelines (2) that recommend starting IVIg in patients with autonomic dysfunction or encephalitis plus acute flaccid paralysis. IVIg has shown to modulate inflammation in EV-71 neurological syndromes by decreasing plasmatic pro-inflammatory cytokines (13). Cytokine removal from plasma could also prevent EV-71 neurological complications (7). Cytokine and other inflammatory humoral factors can be removed with PEX. By this procedure, blood from the patient is passed through a medical device that separates and removes plasma, replacing it with albumin, or donor's plasma. This procedure is safe when performed in critically ill and pediatric patients at experienced centers (14, 15). Good results have been observed in neurological immune-mediated diseases and post-infectious disorders such as steroid-unresponsive acute disseminated encephalomyelitis and Guillain-Barré syndrome (16, 17).

Although the use of PEX is common in parainfectious immune-mediated disorders, EV-71 treatment guidelines (2) do not include PEX as an alternative therapy in severe patients. According to our observations, severe patients could benefit from PEX therapy. In all three cases PEX seemed to change a deteriorating clinical course. Interestingly, patient 3, primarily treated with PEX, recovered quickly after starting PEX. In our opinion, the natural history of the disease, though frequently benign, does not account for this almost immediate improvement. We understand that our observations are limited by the small sample size, uncontrolled design and the concomitant use of conventional treatments such as methylprednisolone and IVIg that may have contributed to the clinical improvement. However, it is unlikely that clinical trials will be available in EV-71 neurological complications and, thus, case reports and case series provide anecdotal but very valuable information to treat patients with potentially fatal complications.

In conclusion, our observations suggest that PEX is a potential adjuvant therapy in patients with severe EV-71 neurological disease. Clearly, however, larger series are needed to confirm the impact of PEX on the prognosis of the disease, and to assess timing and duration of the therapy.

## Ethics Statement

The patient or their parents provided written informed consent agreeing to undergo treatment and allow the publication of the information that was described in the case report.

## Author Contributions

EP-G, LQ, and ET-V were involved in study conceptualization, data collection, drafting, analysis, and revising the manuscript for intellectual content. MJ, CL, MdC, MB, and IJ were involved in data collection, drafting, and revising the manuscript for intellectual content.

### Conflict of Interest Statement

The authors declare that the research was conducted in the absence of any commercial or financial relationships that could be construed as a potential conflict of interest.

## References

[1] Ooi MH Wong SC Lewthwaite P Cardosa MJ Solomon T. Clinical features, diagnosis, and management of enterovirus 71. Lancet Neurol. (2010) 9:1097–105. 10.1016/S1474-4422(10)70209-X 20965438

[2] Cardosa JF Yeng C A Guide to Clinical Management and Public Health Response for Hand, Foot and Mouth Disease. Geneva, Switzerland: World Health OrganizationWestern Pacific Region (2011). ISBN 978 92 9061 525 5. Available online at: http://www.wpro.who.int/publications/docs/GuidancefortheclinicalmanagementofHFMD.pdf (accessed October 1, 2018)

[3] Teoh HL Mohammad SS Britton PN Kandula T Lorentzos MS Booy R. clinical characteristics and functional motor outcomes of enterovirus 71 neurological disease in children. JAMA Neurol. (2016) 73:300–7. 10.1001/jamaneurol.2015.4388 26785318

[4] Schmidt NJ Lennette EH Ho HH. An apparently new enterovirus isolated from patients with disease of the central nervous system. J Infect Dis. (1974) 129:304–9. 4361245 10.1093/infdis/129.3.304

[5] Casas-Alba D de Sevilla MF Valero-Rello A Fortuny C Garcia-Garcia JJ Ortez C. Outbreak of brainstem encephalitis associated with enterovirus-A71 in Catalonia, Spain (2016): a clinical observational study in a children's reference centre in Catalonia. Clin Microbiol Infect. (2017) 23:874–81. 10.1016/j.cmi.2017.03.016 28344164

[6] European Centre for Disease Prevention and Control Enterovirus Detections Associated With Severe Neurological Symptoms in Children and Adults in European Countries. [Internet] 2016. Available online at: https://ecdc.europa.eu/sites/portal/files/media/en/publications/Publications/01-08-2016-RRA-Enterovirus%2071-Spain%2C%20France%2C%20Netherlands.pdf(Accessed October, 17 2018)

[7] Wang SM Lei HY Liu CC. Cytokine immunopathogenesis of enterovirus 71 brain stem encephalitis. Clin Dev Immunol. (2012) 2012:876241. 10.1155/2012/876241 22956971 PMC3432373

[8] Subdirecció General de Vigilància i Resposta a Emergències de Salut Pública de Catalunya Protocol D'actuació Davant Casos D'enterovirus en Població Pediàtrica. [Internet] 2016. Available from: http://salutweb.gencat.cat/web/.content/home/canalsalut/enterovirus/protoenterovirus.pdf. (Accessed October, 17 2018)

[9] Wang SM Lei HY Huang KJ Wu JM Wang JR Yu CK. Pathogenesis of enterovirus 71 brainstem encephalitis in pediatric patients: roles of cytokines and cellular immune activation in patients with pulmonary edema. J Infect Dis. (2003) 188:564–70. 10.1086/376998 12898444

[10] Shao P Wu X Li H Wu Z Yang Z Yao H. Clinical significance of inflammatory cytokine and chemokine expression in hand, foot and mouth disease. Mol Med Rep. (2017) 15:2859–66. 10.3892/mmr.2017.6324 28447721

[11] Defresne P Meyer L Tardieu M Scalais E Nuttin C De Bont B. Efficacy of high dose steroid therapy in children with severe acute transverse myelitis. J Neurol Neurosurg Psychiatry. (2001) 71:272–4. 10.1136/jnnp.71.2.272 11459911 PMC1737519

[12] Wang SM Lei HY Huang MC Wu JM Chen CT Wang JN. Therapeutic efficacy of milrinone in the management of enterovirus 71-induced pulmonary edema. Pediatr Pulmonol. (2005) 39:219–23. 10.1002/ppul.20157 15635619

[13] Wang SM Lei HY Huang MC Su LY Lin HC Yu CK. Modulation of cytokine production by intravenous immunoglobulin in patients with enterovirus 71-associated brainstem encephalitis. J Clin Virol. (2006) 37:47–52. 10.1016/j.jcv.2006.05.009 16861032

[14] Clark SL Rabinstein AA. Safety of intravenous immunoglobulin and plasma exchange in critically ill patients. Neurol Res. (2015) 37:593–8. 10.1179/1743132815Y.0000000017 25751423

[15] Agarwal S Keller JR Nunneley CE Muscal E Braun MC Srivaths P. Therapeutic Plasma Exchange Use in Pediatric Neurologic Disorders at a Tertiary Care Center: A 10-Year Review. J Child Neurol. (2018) 33:140–5. 10.1177/0883073817749368 29334853

[16] Schwartz J Padmanabhan A Aqui N Balogun RA Connelly-Smith L Delaney M. Guidelines on the Use of therapeutic apheresis in clinical practice-evidence-based approach from the writing committee of the american society for apheresis: the seventh special issue. J Clin Apher. (2016) 31:149–62. 10.1002/jca.21470 27322218

[17] van der Meche FG Schmitz PI. A randomized trial comparing intravenous immune globulin and plasma exchange in Guillain-Barre syndrome. Dutch Guillain-Barre Study Group. N Engl J Med. (1992) 326:1123–9. 10.1056/NEJM199204233261705 1552913

